# A Prospective, Multicentre, Open-Label Single-Arm Exploratory Study to Evaluate Efficacy and Safety of Saroglitazar on Hypertriglyceridemia in HIV Associated Lipodystrophy

**DOI:** 10.1371/journal.pone.0146222

**Published:** 2016-01-20

**Authors:** Alka Deshpande, Harsh Toshniwal, Shashank Joshi, Rajendrakumar H. Jani

**Affiliations:** 1 Grant Medical College & Sir J.J. Group of Hospitals, Mumbai, Maharashtra, India; 2 IDTM Clinic, Ahmedabad, Gujarat, India; 3 Joshi Clinic, 12, Golden Palace, Behind Union Bank of India, Turner Road, Bandra West, Mumbai, India; 4 Clinical R & D, Cadila Healthcare Limited, Zydus Research Centre, Sarkhej-Bavla N.H. No. 8A, Moriaya, Ahmedabad, Gujarat, India; Azienda ospedaliero-universitaria di Perugia, ITALY

## Abstract

**Objective:**

This study was designed to explore the efficacy and safety of saroglitazar 4 mg on hypertriglyceridemia in patients with HIV associated lipodystrophy.

**Methods:**

During this 12-week prospective, multi-centric, open-label, single arm exploratory study, 50 patients were enrolled to receive saroglitazar 4 mg orally once daily in the morning before breakfast. The primary efficacy endpoint was the percent change in triglyceride (TG) levels from baseline to Week 6 and Week 12. The secondary efficacy endpoints were assessment of low-density-lipoprotein (LDL), very-low-density-lipoprotein (VLDL), high-density-lipoprotein (HDL), non-HDL cholesterol, total cholesterol, apo-lipoprotein (Apo) A1, Apo B, and C-peptide and fasting insulin for HOMA beta and HOMA IR. Safety assessment was performed during the study.

**Results:**

Saroglitazar 4 mg significantly decreased the serum TG levels from baseline at Week 6 (percent change: -40.98; 95% CI: -50.82, -31.15) and Week 12 (percent change -45.11; 95% CI: -52.37, -37.86). Reduction in VLDL cholesterol (percent change: -46.33; 95% CI: -52.89, -39.76) and total cholesterol (percent change: 7.37; 95% CI: 1.96, 12.78) was observed at week 12 from baseline. Saroglitazar increased HDL cholesterol (percent change: 34.56, 95% CI: 22.22, 46.90), Apo A1 (percent change: 33.16; 95% CI: 18.69, 47.63) and Apo B (percent change: 10.55, 95% CI: 2.86, 18.25) levels at week 12 from baseline. Saroglitazar treatment led to increase in the C-peptide (percent change: 59.42, 95% CI: 48.78, 70.06), fasting insulin levels (percent change: 47.10; 95% CI: 38.63, 55.57), HOMA of beta cell function for C-peptide (percent change: 71.67; 95% CI: 39.09, 104.26) and HOMA of insulin resistance for C-peptide (percent change: 58.29, 95% CI: 46.74, 69.83) at week 12 from baseline. Saroglitazar treatment was safe and well tolerated in this study.

**Conclusion:**

Overall, the observed changes in lipid profile after 12 weeks of saroglitazar treatment were in the direction of improvement in patients with HIV associated lipodystrophy.

**Trial Registration:**

Clinical Trial Registry of India Phase II/CTRI/2010/091/000107

## Introduction

The lifelong exposure to highly active antiretroviral therapy (HAART) is associated with a significant risk of long term metabolic adverse effects including lipodystrophy, insulin resistance, hyperlipidemia and increased cardiovascular morbidity in patients with HIV [1]. The prevalence of HIV lipodystrophy affects up to 20%-80% of patients receiving ART depending upon the population and the study [2]. Patients with this condition develop a pattern of redistribution in body fat characterised by peripheral fat loss (facial and limb lipoatrophy) and central fat accumulation [2, 3–6]. The recognised metabolic disturbances include abnormalities in both triglyceride (TG) and cholesterol levels in the blood. Resultant cholesterol abnormalities tend to include elevation of low density lipoprotein (LDL) and very low density lipoprotein (VLDL) concentrations [7]. The association between HIV infection, antiretroviral therapy (ART), and coronary heart disease (CHD) has been reported in several studies [3]. Although linked to antiretroviral therapy, the exact etiology of HIV lipodystrophy remains unclear.

A variety of drugs have been studied to determine their influence on lipid profiles in patients suffering from HIV associated lipodystrophy due to HAART. However, no single therapy is able to reach desirable clinical end point for HIV associated lipodystrophy [7].

Saroglitazar is a novel predominately peroxisome proliferator-activated receptors (PPARα) agonist and moderate PPARγ receptor agonist. Zydus Research Centre, a research wing of Cadila Healthcare Limited, has carried out extensive pre-clinical studies with saroglitazar using various *in-vitro* and animal models wherein, EC_50_ of PPARα: PPARγ was >300; favourably modulated the lipid & glucose profile. Phase I [8], and other Phase II studies of saroglitazar demonstrated favourable effects in modulating lipid profile, glucose profile and ameliorate insulin resistance (unpublished data). The efficacy of saroglitazar in reducing triglyceride has been well established in several Phase III studies [9, 10].

Thus, the present study was designed to explore the safety and efficacy of saroglitazar 4 mg on hypertriglyceridemia in patients with HIV associated lipodystrophy.

## Materials and Methods

### Study design and participants

This was a 12 weeks prospective, multi-centric, open-label, single arm exploratory study designed to explore the safety and efficacy of Saroglitazar 4 mg on hypertriglyceridemia in 50 patients with HIV associated lipodystrophy at 2 investigational sites in India.

Patients with following inclusion criteria were enrolled in trial: male or female aged 18–65 years; diagnosis of HIV1 and on HAART for at least 18 months; on stable ART regimen for at least 8 weeks prior to inclusion in the study and ART regimen not expected to change in next 3 months; patient clinically diagnosed as HIV lipodystrophy (at least 1 moderate or severe lipodystrophy feature identified by doctor and patient, except isolated abdominal obesity); triglycerides level >200 to 500 mg/dL; CD4 count of >50/mm^3^ and patient who had given written informed consent for participation in the trial.

Patients were excluded if they were on insulin and/or glitazone/glitazar therapy; pregnancy and lactation; history of gall stones, cardiac failure, alcohol and/or drug abuse; history of allergy, sensitivity or intolerance to the study drug and its formulation ingredients; active opportunistic infection in last three months; history of malignancy or active neoplasm; any active hormonal disease and/or hormonal treatment that could affect the outcomes of interest such as clinically overt hypo/hyperthyroidism, hypogonadism, hypercortisolism, or treatment with steroids or growth hormone; hemoglobin below 9 g/dL or total leucocyte count below 1000/mm^3^ or platelet count below 50,000/mm^3^; history of myopathies or evidence of active muscle diseases or CPK ≥10 times upper limits of normal (ULN); history of active liver disease or hepatic dysfunction demonstrated by aspartate aminotransferase (AST) and alanine aminotransferase (ALT) ≥2.5 times of upper limits of normal or bilirubin more than 2 times UNL; renal dysfunction (serum creatinine >2 mg/dL) and participated in any other clinical trial in past 3 months.

Patients satisfying inclusion and exclusion criteria were enrolled from 21 August 2010 (first patient first visit) to 14 September 2010 (last patient first visit). The last patient last visit for the study was 09 December 2010. The study was Good Clinical Practice compliant and initiated after obtaining the approvals from the Drug Controller General of India (DCGI) and registration of the trial with Clinical Trial Registry of India (Phase II/CTRI/2010/091/000107). The trial was submitted to CTRI on 02 February 2010 with a reference number REFCTRI/2010/000107; though the CTRI number was issued on 09 September 2010.

The Independent Ethics Committee (IEC)- Aditya, Ahmedabad, date of approval 25 June 2010 and the Institutional Ethics Committee of Grant Medical College and Sir J.J. Group of Hospital, Mumbai, date of approval 03 August 2010 had reviewed and approved the study (S2 and S3 Files). The authors confirm that all ongoing and related trials for this drug/intervention are registered with Clinical Trial Registry of India (http://ctri.nic.in/Clinicaltrials/pmaindet2.php?trialid=1322 and http://www.ctri.nic.in/Clinicaltrials/pmaindet2.php?trialid=9538). The written informed consent was obtained from each participant before initiation of any study related procedure.

### Procedure

After screening the eligible subjects were enrolled in the study and baseline characteristics were recorded. During this 12 week study patients were seen on Week 2, Week 6 and Week 12. Patients received study medication on every visit and were advised to take saroglitazar 4 mg orally once daily in the morning before breakfast for a period of 12 week.

The primary efficacy endpoint was to assess the percent change in triglyceride levels from baseline to Week 6 and Week 12. The secondary efficacy endpoints were assessment of LDL, VLDL, HDL, Non HDL cholesterol, total cholesterol, Apo A1, Apo B, and C-peptide and fasting insulin for HOMA beta and HOMA IR from baseline to week 6 and week 12.

Safety variables were assessed at week 2, week 6 and week 12 which includes vital and physical examination, laboratory investigation and adverse events (AEs) assessment. Adverse events were graded on severity (i.e., mild, moderate, severe) and coded using the Medical Dictionary for Regulatory Activities (MedDRA, Version 14).

### Statistical analysis

Data were analyzed using SAS Software version 9.1 (SAS Institute Inc., Cary, NC, USA). The demographic and baseline characteristics were summarized. For continuous measurements such as age; the mean & standard deviation (SD) were tabulated. For categorical measurements such as gender, the frequencies were computed. Enrolment Visit 1 (Week 0) is considered as baseline and used for deriving the percent change. The primary efficacy variable is the percent change in triglyceride at Week 6 and Week 12 compared with baseline.

The percent change from baseline was determined as:

$$Percent\;Change=\frac{\left\{\left(Week\;6\;or\;Week\;12\right)\right\}-\;Baseline}{Baseline}*100$$

Percent Change from baseline for efficacy variables were analyzed using analysis of covariance (ANCOVA) with respective baseline value as covariate. Least-square means (LSM) and 95% confidence intervals were evaluated from the ANCOVA. The data of percent changes were assumed as normally distributed. All other safety laboratory parameter was analyzed using same statistical methods.

## Results

**Fig 1:**
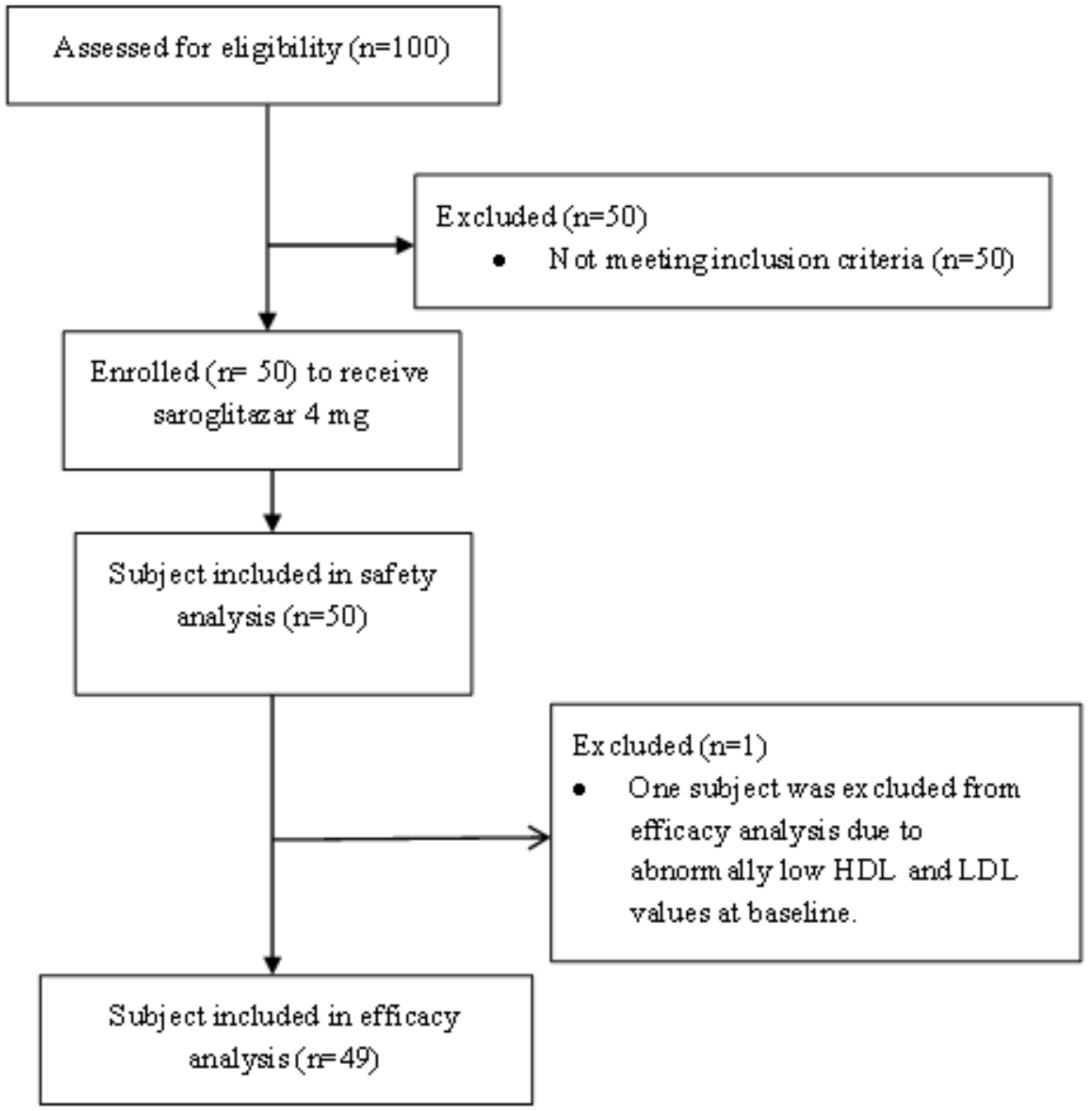
Flow diagram of study participation.

The demographic details and flow diagram of participation is provided in Table 1 and Fig 1, respectively. Of 50 patients, 49 patients were assessed for efficacy analysis. A patient was excluded from analysis due to low levels of HDL and LDL at Visit 1. Among the enrolled patients 64% (n = 32) were male and 36% (n = 18) were female. Overall mean age was 40.26±7.13 years with mean body weight (kg) of 52.72±7.86 and BMI (kg/m^2^) of 20.86±2.73. The median duration of ART at the time of enrollment was approx. 3 years. About 58% patients were on stavudine, lamivudine and nevirapine regimen followed by 28% of patients were on zidovudine, lamivudine and nevirapine regimen, 12% of patients were on stavudine, lamivudine and efavirenz regimen, and 2% patients were ontenofovir, emtricitabine and nevirapine regimen.

**Table 1:** Summary of demographic and baseline characteristics (Safety population). Abbreviations: BMI = body mass index; cms = centimeter; kg = kilogram; m = meter; N = number of subjects; n = number of subjects with non-missing values, SD = standard deviation; yrs = years;

Variable	Statistic	Saroglitazar 4 mg (N = 50)
Gender		
Female	n (%)	18 (36%)
Male	n (%)	32 (64%)
Age (yrs)	Mean±SD	40.26±7.13
Weight (kgs)	Mean±SD	52.72±7.86
Height (cm)	Mean±SD	158.82±5.83
BMI (kg/m^2^)	Mean±SD	20.86±2.73
Median duration of ART therapy (yrs)	Median	3.00
Absolute CD4 count (/uL)	Mean±SD	521.69±275.20

**Fig 2:**
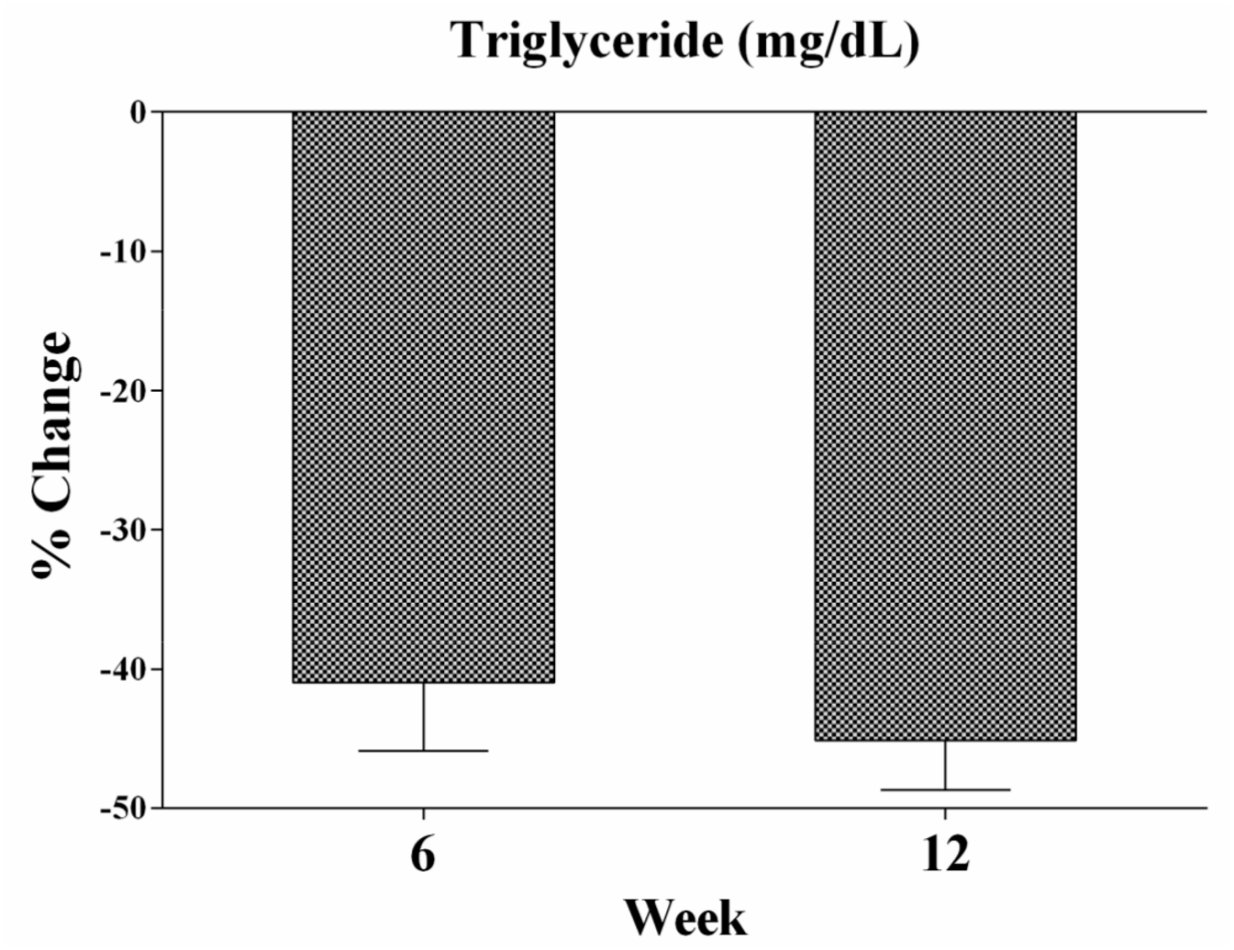
Percentage change in triglyceride level following saroglitazar 4 mg treatment. At week 12 saroglitazar treatment led to increase in the C-peptide (percentage change: 59.42, 95% CI: 48.78, 70.06) and fasting insulin levels (percentage change: 47.10; 95% CI: 38.63, 55.57) when compared with baseline; however, it was with in normal range. Remarkable increase was observed in HOMA of beta cell function for C-peptide (percent change: 71.67; 95% CI: 39.09, 104.26) and HOMA of insulin resistance for C-peptide (percent change: 58.29, 95% CI: 46.74, 69.83) at week 12 from baseline.

Saroglitazar 4 mg treatment demonstrated a statistically significant reduction in serum TG levels from baseline to Week 6 and Week 12 (percent change: -40.98; 95% CI: -50.82, -31.15 and percent change-45.11; 95% CI: -52.37, -37.86) respectively. The summary of efficacy endpoints is provided in Table 2 and Fig 2. After 12 weeks of saroglitazar 4 mg treatment, there was also reduction in VLDL cholesterol (percent change: -46.33; 95% CI: -52.89, -39.76) and total cholesterol (percent change: 7.37; 95% CI: 1.96, 12.78) compared to baseline. Saroglitazar 4 mg treatment also resulted in improvements of lipid parameters. There was increase in HDL cholesterol (percent change: 34.56, 95% CI: 22.22, 46.90), Apo A1 (percent change: 33.16; 95% CI: 18.69, 47.63) and Apo B (percent change: 10.55, 95% CI: 2.86, 18.25) levels compared to baseline at week 12.

**Table 2:** Summary of efficacy endpoints at Week 6 and Week 12 (N = 49). Abbreviations: LSM = least square mean; SD = standard deviation; SE = standard error; N = number of subjects; Note: *—indicates significant (p <0.0001) and calculated for primary endpoints.

Variables (Units)	Time points (Mean±SD)			Percentage change from week 0 to week 6		Percent Change from week 0 to week 12	
	Week 0	Week 6	Week 12	LSM±SE	95% Confidence Interval	LSM±SE	95% Confidence Interval
**Primary Endpoint**							
**Triglyceride (mg/dL)**	301.68±86.99	172.81±106.30	166.97±89.17	-40.98±4.89*	(-50.82, -31.15)	-45.11±3.60*	(-52.37, -37.86)
**Secondary Endpoint**							
Low density lipoprotein cholesterol (mg/dL)	124.29±34.52	123.98±37.09	130.37±49.44	2.76±3.28	(-3.83, 9.36)	6.53±4.86	(-3.25, 16.30)
Very low density lipoprotein cholesterol(mg/dL)	61.44±19.14	34.56±21.26	33.39±17.83	-41.60±4.88	(-51.43, -31.77)	-46.33±3.26	(-52.89, -39.76)
High density lipoprotein (HDL) cholesterol (mg/dL)	35.27±7.85	44.44±14.04	46.14±14.84	29.92±5.73	(18.39, 41.45)	34.56±6.13	(22.22, 46.90)
Total cholesterol (mg/dL)	195.17±45.31	197.45± 46.20	207.44±53.29	2.45±2.11	(-1.79, 6.69)	7.37±2.69	(1.96, 12.78)
Non HDL cholesterol (mg/dL)	159.90±44.02	153.01±45.62	161.71±53.42	-2.37±2.77	(-7.95, 3.20)	2.41±3.26	(-4.16, 8.97)
Apolipoprotein A1 (mg/dL)	146.58±29.78	178.26±59.22	190.46±67.84	29.59±6.40	(16.72, 42.46)	33.16±7.19	(18.69, 47.63)
Apolipoprotein B (mg/dL)	79.92±20.32	76.93±21.60	86.93±26.76	-1.99±2.97	(-7.97, 3.99)	10.55±3.82	(2.86, 18.25)
C-peptide (ng/mL)	2.16±1.08	2.94±1.97	2.94±0.82	44.24±9.43	(25.27, 63.21)	59.42±5.29	(48.78, 70.06)
HOMA of beta cell function for C-peptide	132.04±64.82	162.96±80.46	180.26±52.49	68.25±25.58	(16.79, 119.71)	71.67±16.20	(39.09, 104.26)
HOMA of insulin resistance for C-peptide	1.59±0.82	1.86±0.77	2.15±0.62	27.87±4.22	(19.37, 36.37)	58.29±5.74	(46.74, 69.83)
Insulin (fasting) μu/Ml	9.21±6.26	10.42±5.74	11.40±4.45	23.71±3.55	(16.57, 30.86)	47.10±4.21	(38.63, 55.57)
HOMA of beta cell function for insulin	107.82±52.85	136.41±76.00	137.56±46.11	52.50±14.94	(22.42, 82.57)	45.64±6.22	(33.11, 58.16)
HOMA of insulin resistance for insulin	1.21±0.80	1.40±0.71	1.46±0.55	29.10±3.94	(21.18, 37.03)	42.65±3.79	(35.02, 50.28)

The change in anthropometry parameters were also assessed following 12 week of saroglitazar treatment. Remarkable changes were observed in triceps, biceps and skin fold thickness. The Summary of anthropometry parameters is presented in Table 3.

**Table 3:** Summary of anthropometry parameters. Abbreviations: BMI = body mass index; kg = kilogram; m = meter; mm = millimeter; N = number of subjects; SD = standard deviation;

Parameters	Saroglitazar 4 mg (N = 50)	
	Week 0 (Mean±SD)	Week 12 (Mean±SD)
Triceps (mm)	9.54±5.60	10.38±5.96
Biceps (mm)	6.16±6.91	7.18±7.63
Subscapular (mm)	10.12±4.89	10.50±5.43
Skin fold thickness (mm)	12.64±5.95	13.16±5.52
Body weight (kg)	52.72 ±7.86	52.78 ± 7.69
BMI (kg/m^2^)	20.86±2.73	20.87±2.48

Overall, saroglitazar 4 mg was safe and well tolerated by patients with hypertriglyceridemia in HIV associated lipodystrophy. There were no deaths during this study. One SAE of severe stomach pain was reported during the study which was resolved completely and it was considered probably related to the study drug by the investigator. The overall incidence of AEs was less than 10%. Adverse events from the gastrointestinal disorder system organ class (SOC) (constipation and abdominal pain) were the most common, which were of either mild or moderate in intensity. There were no clinically significant changes in the laboratory parameters, vital signs or physical examination findings during the study. Table 4 shows the summary of safety parameters.

**Table 4:** Summary of safety parameters (N = 50). Abbreviations: LSM = least square mean; SD = standard deviation; SE = standard error; N = number of patients;

Laboratory variables (Unit)	Week 0 (Baseline)	Week 12 (End of the study)	Percent Change from baseline to week 12	
	Mean±SD	Mean±SD	LSM±SE	95% Confidence Interval
Fasting plasma glucose (mg/dL)	95.18±36.08	90.54±35.26	-3.47±1.73	(-6.95, 0.01)
Blood urea nitrogen (mg/dL)	9.29±3.11	9.38±2.60	7.71±4.35	(-1.03, 16.45)
Creatinine (mg/dL)	0.73±0.25	0.75±0.20	8.58±3.61	(1.32, 15.85)
Estimated glomerular filtration rate (eGFR) (mL/min)	102.55±27.90	97.44±26.78	-0.16±3.65	(-7.50, 7.18)
Total bilirubin (mg/dL)	0.97±4.19	0.33±0.11	-5.46±6.94	(-19.42, 8.50)
Alanine aminotransferase (U/L)	31.90±15.80	26.88 ±13.70	-5.20±6.53	(-18.34, 7.94)
Aspartate aminotransferase (U/L)	30.02±12.34	36.16±20.22	28.28±9.39	(9.40, 47.16)
Gamma-glutamyl transpeptidase(U/L)	101.10±61.16	60.43±32.25	-34.53±3.45	(-41.47, -27.58)
Alkaline phosphates (ALP) (U/L)	105.17±59.48	66.96± 42.30	-35.21±2.12	(-39.47, -30.94)
Protein total (g/dL)	8.14±0.62	8.12±0.59	-0.01±0.75	(-1.51, 1.50)
Albumin (g/dL)	4.65±0.27	4.83±0.30	4.10±0.85	(2.39, 5.80)
Globulin (g/dL)	3.49±0.63	3.29±0.68	-5.65±1.28	(-8.23, -3.08)
A/G Ratio	1.37±0.22	1.52±0.30	11.10±1.66	(7.77, 14.44)
Creatine phosphokinase (Total) U/L	173.99±129.99	217.79±227.42	33.05±12.73	(7.44, 58.65)
Haemoglobin (gm/dL)	13.25±1.74	13.07±1.84	-1.24±0.98	(-3.21, 0.73)
Total red blood cell count (10^6/ uL)	3.86±0.62	3.66±0.62	-5.12±1.09	(-7.32, -2.93)
Total platelet count (10^3 / uL)	202.84±57.04	236.62±76.09	18.39±3.78	(10.79, 25.99)
Total leucocyte count (TLC) (10^3 / uL)	6.70±1.96	6.64±2.24	0.20±3.21	(-6.27, 6.66)

## Discussion

A variety of drugs have been studied to determine their influence on lipid profiles in patients suffering from HIV associated lipodystrophy due to HAART. The use of fibrates as first-line therapy for hypertriglyceridemia is recommended. This is supported by the various studies that demonstrated that fibrates effectively lower triglycerides and in certain cases improves total cholesterol, LDL and HDL. In addition, it appears that the incidence of side effects was not higher in patients with HIV than those observed in patients without HIV [11–21].

The thiazolidinediones, which are PPAR-γ agonists, are true insulin sensitizers affecting insulin action in peripheral tissues, such as skeletal muscle and adipose tissue [22]. There has been much interest in their use in the treatment of the lipodystrophy syndrome associated with ARTs. It was expected that an increase in insulin sensitivity will improve lipid profile; however, some trials have shown negative effects of these medications on lipid endpoints [23].

Recent research publications have reported the use of two lipid-lowering class of drugs, statins and fibrates, antiretroviral switching strategies and use of insulin-sensitizing drugs in the management of the HIV lipodystrophy. However, no therapy is able to reach desirable clinical end point for HIV associated lipodystrophy [24].

The National Cholesterol Education Program (NCEP) identified metabolic syndrome, LDL, triglyceride and HDL as important targets. For patient with very high triglycerides the NCEP recommends initially targeting therapy to lower triglycerides before turning to LDL to further assess CHD risk [24,25]. In contrast, 2013 American College of Cardiology (ACC)/American Heart Association (AHA) identified four risk groups that need to be prescribed either moderate or high intensity statin therapy, regardless of their baseline LDL-C levels and without aiming for a particular pre-defined LDL-C target. This guideline cited lack of evidence to support the use of non-statin cholesterol lowering drugs, either in combination with statins or as mono-therapy in statin-intolerant patients [26]. However, a Phase III study of saroglitazar in comparison with placebo in patients with diabetic dyslipidemia who were not controlled with atorvastatin therapy reported favorable improvement in lipid profile including triglyceride, LDL cholesterol, non-HDL-C, VLDL, total cholesterol, and fasting plasma glucose [9].

In the present study saroglitazar showed statistically significant reduction in serum TG levels from baseline to Week 12 following saroglitazar 4 mg therapy once daily in the patients of HIV associated lipodystrophy.

There was also reduction in serum VLDL levels from baseline to Week 12. Abnormalities of glucose homeostasis such as insulin resistance affect substantial number of HIV infected patients receiving HAART. Insulin resistance may be due to severe change in fat redistribution as well as from direct effects of antiretroviral drugs. [27]. Stavudine association with greater insulin resistance in HIV-infected patients compared to other NRTIs is well known. In the current study majority of the patients enrolled were on stavudine regimen. There were increase in C-peptide levels, HOMA of beta-cell function derived from C-peptide, HOMA of insulin resistance derived from C-peptide, insulin (fasting) levels, HOMA of beta-cell function derived from insulin and HOMA of insulin resistance derived from insulin at week 6 and 12 following administration of saroglitazar 4 mg. Increasing level of insulin and HOMA IR are associated with HAART and risk of diabetes; however, surprisingly there was an improvement in HOMA of beta-cell function derived from insulin [28].

There was increase in the HDL cholesterol levels, Apo A1 levels; however, decrease in the LDL and Non-HDL cholesterol levels was not significant from baseline following 12 weeks of saroglitazar 4 mg treatment.

An initial slight reduction was observed in the serum Apo B levels at Week 6. This was followed by an increase in Apo B levels from baseline following administration of saroglitazar 4 mg at week 12, which was significant. It has been reported that HIV-positive patients receiving antiretroviral therapy have increased secretion and decreased clearance of VLDL particles [29] increased synthesis [30] and reduced catabolism of apolipoprotein B [31] the protein backbone of atherogenic lipoproteins, and a diminished lipoprotein lipase activity [32]. As a downstream effect of these underlying mechanisms, and in addition to frank hypertriglyceridemia, an increased level of proatherogenic remnant lipoprotein levels has been noted in HIV-positive patients undergoing HAART [33,34]. Eventually, two confirmatory Phase III studies of saroglitazar in diabetic dyslipidemia showed significant reduction in apolipoprotein B [9,10].

Overall, saroglitazar 4 mg was safe and well tolerated by patients having hypertriglyceridemia in HIV associated lipodystrophy in this study. There was no death reported during the study. An SAE of severe stomach pain was reported which was completely resolved and considered probably related to the study drug by the investigator. The overall incidence of AEs was <10%. Adverse events from the GI disorder SOC (constipation and abdominal pain) were the most common AEs, which were of either mild or moderate in intensity.

There were no clinically significant changes in the laboratory parameters, vital signs or physical examination findings; however, some significant changes were observed in lab parameters from baseline and in anthropometry parameters.

It has been reported that the increased level of CPK is associated with HIV related medication. Moreover, the risk of myopathy increases with lipid lowering treatment [35]. In present study the patients were also observed with high level of CPK at baseline and also at the end of study.

This study cannot be generalized as this has several limitations. The sample size was too small and duration was too short to assess long term safety of the drug, in addition this study was open label without control group; however, this small exploratory trial has provided a base for the current ongoing double-blind, randomized, placebo controlled Phase III study (CTRI/2014/08/004885; http://www.ctri.nic.in/Clinicaltrials/pmaindet2.php?trialid=9538) where saroglitazar efficacy and safety will be assessed over a period of 52 weeks in patients with HIV associated lipodystrophy. The primary endpoint is change in the visceral adipose tissue after 52 weeks of treatment. This trial will provide further understanding of saroglitazar efficacy and safety in patients with HIV associated lipodystrophy.

## Supporting Information

S1 File This is the protocol. (PDF)

S2 File This is the Ethics Committee Approval Letter 1. (PDF)

S3 File This is the Ethics Committee Approval Letter 2. (PDF)

S4 File This is CONSORT 2010 checklist. (PDF)
