# Supplementary material for: A Prospective, Multicentre, Open-Label Single-Arm Exploratory Study to Evaluate Efficacy and Safety of Saroglitazar on Hypertriglyceridemia in HIV Associated Lipodystrophy
Source: PLoS One. 2016 Jan 20;11(1):e0146222. doi: 10.1371/journal.pone.0146222 (PMC4720399; doi:10.1371/journal.pone.0146222)
Supplement: S1 File — (PDF) [file pone.0146222.s001.pdf]

	Zydus Cadila
**Clinical Trial Protocol**	Cadila Healthcare Limited

**CHL Trial No.:**	ZYH1.09.003.01 .PROT
**Investigational: product(s)**	ZYH1
**Title**:	A prospective, multi-centric, open-label, single arm study to evaluate the safety and efficacy of 4mg of ZYH1 in hypertriglyceridemia in HIV associated lipodystrophy
**Clinical Phase:**	II
**Sponsor**	Cadila Healthcare Limited, India
**Study Director: (Sponsor)**	Dr. R. H. Jani, Ph.D. (Medical)<br>Senior Vice President, Clinical R&D, Cadila Healthcare Limited.
**Sponsor's Medical Expert:**	Dr. Dhiraj Gambhire, MD (Medicine), Scientist<br>Dr. Bhavna N. Mulgaonkar, MBBS, DPB, Scientist
**Date of Protocol:**	22.06.2010
**Planned Dates of Trial:**	30.06.2010
**Confidential**	© Cadila Healthcare Limited<br>This protocol is the property of Cadila Healthcare Limited and may not - in full or in part - be retrieved, passed on, reproduced, published or otherwise use without the express permission of Senior Vice President, Clinical R&D, Cadila Healthcare Limited.

CLINICAL TRIAL PROTOCOL SUMMARY

**Name of Sponsor:**		Cadila Healthcare Limited	
**Name of Investigational product:**		ZYH1 4mg	
**Name of active ingredient of investigational product**		ZYH1	
**Name of the comparator drug**		NA	
**Name of active ingredient of comparator product**		NA	
**Protocol synopsis date:** 09.03.2009	**Trial Number:** ZYH1.09.003.01.PROT	**Planned Study period:** 12 weeks	**Study Duration:** 05 months
**Title of study:**<br><br>A prospective, multi-centric, open-label, single arm study to evaluate the safety and efficacy of 4mg of ZYH1 in hypertriglyceridemia in HIV associated lipodystrophy.			
**Clinical phase:** Phase II			

Objectives:
To evaluate the safety and efficacy of 4mg of ZYH1 in hypertriglyceridemia in HIV associated lipodystrophy.

Primary Efficacy (Time frame 6 week and 12 week):

- TG

Secondary Efficacy (Time frame 6 week and 12 week):

- LDL
- VLDL
- HDL
- Total cholesterol
- Non HDL Cholesterol (Measured value)
- Apo (a)
- Apo B
- Fasting insulin and C-peptide for HOMA beta & IR

Criteria for Safety:

- General and Systemic Clinical Examination: cardiovascular system (CVS), respiratory system (RS), gastrointestinal system (GI), central nervous system (CNS) etc.
- Laboratory Investigations: Complete blood count (CBC), aspartate transaminase (AST), alanine transaminase (ALT), alkaline phosphatase (ALP), total bilirubin, γ- glutamyl transpeptidase (GGT), Serum proteins, Blood urea nitrogen (BUN), Serum creatinine, and Creatinine phosphokinase (CPK).
- Frequency and severity of adverse events (AEs) for all subjects enrolled will be recorded. All AEs, will be classified using
    - causality
    - severity
    - seriousness

Methodology:

It is an interventional, single arm, safety and efficacy study to explore the effects of ZYH1 on hypertriglyceridemia in HIV associated lipodystrophy.

- Duration of the treatment: 12 weeks
- Subjects should be clinically diagnosed as HIV Lipodystrophy. Lipodystrophy will be diagnosed by at least 1 moderate or severe lipodystrophy feature identified by doctor and patient, except isolated abdominal obesity.
- Subjects should have confirmed diagnosis of HIV 1 and on highly active antiretroviral therapy (HAART) for at last 18 months.
- HAART regimen shall not be expected to change in next 3 months.
- Triglyceride level should be ranging between 200 to 500 mg%.
- Such subjects will be invited to participate in this study.
- Evaluation for safety and efficacy will be done as per the activity chart in Table 1.

Study Schedules:

Informed consent will be obtained before any trial related activity.

1)Visit 1, Screening/Enrolment Visit [Week ≥ -1 to 0]

- Subjects will be screened for the inclusion and exclusion criteria and those qualifying will be invited to participate in the study.
- Clinical evaluation will be done for baseline characteristics and anthropometry.
- After Clinical evaluations all baseline safety and efficacy parameters will be recorded as per Table 1.
- All laboratory investigations will be carried out after an overnight fasting.

- Enrolled Subjects will receive a study medication for next two weeks

2)Visit 2, [Week 2]

Subjects will be clinically examined and given the study medications for next four weeks and also safety parameters will be assessed as per Table 1.
Empty strips will be collected back by the investigators for compliance check.

3)Visit 3 [Week 6]
Subjects will be clinically examined and given the study medications for next 6 weeks and also safety and efficacy parameters will be assessed as per Table 1.
Empty strips will be collected back by the investigators for compliance check.

4)Visit 4 [Week 12]
Subjects will be clinically examined and safety and efficacy parameters will be assessed as per Table 1.
Empty strips will be collected back by the investigators for compliance check.

Unscheduled visits are allowed any time during the study for any untoward effect. Such visits will be recorded in the case record forms (CRFs).

If further investigations are required in case of any AE, investigator will assess the AE and take necessary action, if required. Subjects will be advised to contact the investigator for any complaints within the next two weeks.

During the above period, if any subject misses the drug administration up to 3 days, it will not be considered drop-out or protocol deviation.

During the 12 week program, a designated person from the centre will preferably interview the subject for his/her general health, telephonically.

Table 1: Visit and Investigation Schedule

Activity	Screening / Enrolment Visit 1 (Wk ≥-1 to 0)	Visit 2 (Week 2)	Visit 3 (Week 6)	Visit 4 (Week 12)
Demographics	✓			
Inclusion / Exclusion criteria	✓			
Informed Consent	✓			
Medical History	✓	✓	✓	✓
Vital Signs & Physical Examination	✓	✓	✓	✓
Efficacy studies:<br>Laboratory:<br>1. Triglyceride<br>2. LDL<br>3. VLDL<br>4. HDL<br>5. Total cholesterol<br>6. Non HDL Cholesterol<br>7. Apo(a)<br>8. Apo B<br>9. Fasting insulin and C-peptide for HOMA beta & IR	✓		✓	✓
Laboratory studies(safety)<br>1. CBC<br>2. LFT<br>3. RFT<br>4. CPK	✓	✓	✓	✓
ECG	✓			✓
Pregnancy test for female subjects (advise for contraception)	✓			
Dispensing of Study Medication	✓	✓	✓	
Study Medication Tablet Count		✓	✓	✓
Recording of Adverse Events		✓	✓	✓
Global Tolerability Assessments				✓
Study Completion				✓

Protocol Deviations:

During this study following will be considered as serious protocol deviations:

(1) Failure to report within +/- 4 days of the planned visits

(2) Failure to take medicine for more than 3 consecutive days
(3) Self medication of prohibited medicines by this protocol
(4) Serious non compliance with regulatory or Good Clinical Practice (GCP) guidelines
(5) Violations of inclusion and exclusion criteria on screening visit / enrolment visit.

Subject Withdrawal Criteria:

(1) Subject who develops any serious/severe diseases which requires admission in intensive care unit (ICU) or interventional surgery, even if it is not related with investigational drugs
(2) Any subject found to have entered the study in violation of this protocol or if the subject is uncooperative during the study.
(3) If it is felt in Investigator's / Medical Expert's opinion that it is not in the subject's best interest to continue.
(4) Patient requiring change in HAART will be excluded from the study.
(5) Subject's lack of compliance to the study protocol
(6) Any subject who requires the use of an unacceptable concomitant medication.
(7) Any subject who wishes to withdraw his / her consent for participation in the study.

Concomitant Medication:

(1) Treatment of common illness will be allowed which shall be documented in the case report form (CRF).
(2) Treatment of opportunistic infections will be allowed during study period. Same will be recorded in the CRF.

Restricted Medications:

1) Other lipid lowering drugs will not be allowed during the study.
2) Drugs affecting insulin resistance will not be allowed during the study.
3) Other drugs claimed for treatment of lipodystrophy will not be allowed during the study.
4) Change in antiteroviral treatment is not allowed during the study. If changes are necessary such patient will be withdrawn from study.

**No. of subjects in treatment arm: Total:**	50

Criteria for inclusion/exclusion:

INCLUSION CRITERIA (S):

- Age 18- 65 years
- Subjects should have confirmed diagnosis of HIV 1 and on highly active antiretroviral therapy (HAART) for at last 18 months.
- On stable ART regimen for at least 8 weeks prior to inclusion in the study and ART regimen not expected to change in next 3 months.
- Subjects clinically diagnosed as HIV Lipodystrophy (at least 1 moderate or severe Lipodystrophy feature identified by doctor and patient, except isolated abdominal obesity)
- Triglycerides between 200 – 500 mg%
- Subject has given informed consent for participation in this trial
- Subjects whose CD4 count is > 50/mm^3

EXCLUSION CRITERIA(S):

- Subjects on insulin and/or glitazone / glitazar therapy
- Pregnancy and lactation
- Subjects with history of active liver disease or hepatic dysfunction demonstrated by aspartate aminotransferase (AST) and alanine aminotransferase (ALT) ≥2.5 times of upper limits of normal or Bilirubin more than 2 times UNL
- Renal dysfunction (serum creatinine > 2 mg%)
- Subjects with history of gall stone.
- Subjects with history of Cardiac failure.
- Subjects with history of myopathies or evidence of active muscle diseases or CPK ≥10 times UNL
- Subject with history of alcohol and/or drug abuse
- History of allergy, sensitivity or intolerance to the study drugs and their formulation ingredients.
- History of active Opportunistic infection in last three months.
- History of malignancy or active neoplasm.
- Any active hormonal disease and/or hormonal treatment that may affect the outcomes of interest such as clinically overt hypo/hyperthyroidism, hypogonadism, hypercortisolism, or treatment with steroids or growth hormone.
- Hemoglobin below 9 g/dl or Total leukocyte count below 1000/mm^3 or Platelet count below 50,000/mm^3
- Participation in any other clinical trial in past 3 months

**Test product:**<br>**Dose:**<br>**Mode of administration:**	ZYH1<br>4mg once daily (OD)<br>Oral
**Reference therapy:**	NA
**Duration of treatment :**	12 weeks
**Criteria for efficacy**	**Primary Efficacy (Time frame 6 and 12 weeks):**<br>• TG<br><br>**Secondary Efficacy (Time frame 6 and 12 weeks):**<br>• LDL<br>• VLDL<br>• HDL<br>• Total cholesterol<br>• Non HDL Cholesterol (Measured value)<br>• Apo (a)<br>• Apo B<br>• Fasting insulin and C-peptide for HOMA beta & IR
**Criteria for safety:**	• General and Systemic Clinical Examination: cardiovascular system (CVS), respiratory system (RS), gastrointestinal system (GI), central nervous system (CNS) etc.<br>• Laboratory Investigations: Complete blood count (CBC), aspartate transaminase (AST), alanine transaminase (ALT), alkaline phosphatase (ALP), total bilirubin, γ- glutamyl transpeptidase (GGT), Serum proteins, Blood urea nitrogen (BUN), Serum creatinine and Creatinine phosphokinase (CPK)<br>• Frequency and severity of adverse events (AEs) for all subjects enrolled will be recorded. All AEs, will be classified using<br>▪ causality<br>▪ severity<br>▪ seriousness

Statistical methods:

The SAS® package (SAS® Institute Inc., USA, and Version 9.1.3) will be used for statistical evaluation.

The demographic and baseline characteristics will be summarized. For continuous measurements such as age, the mean, median, standard deviation and range will be tabulated. For categorical measurements such as gender, the frequencies will be computed.

The primary efficacy variable is the Reduction in Triglyceride (TG) at Week 6 and 12 of the treatment period compared with baseline. The change from baseline will be determined as the difference between the means for the treatment period (Week 6 and12) and the baseline.

For the efficacy endpoints, treatment effect will be evaluated using an analysis of variance (ANOVA) model. Treatment effects will be estimated using the least-square means and 95% confidence intervals (CIs) from the ANOVA model. Statistical significance will be defined as a two-sided p-value <0.05.

All other secondary endpoints would be analysed using appropriate statistical methods.<br>Intent-to-treat (ITT) and/or Per Protocol (PP) analysis will be carried out for the study trial. The PP analysis will be considered definitive while the ITT analysis will be considered supportive during the trial analysis. All the subjects enrolled in the study will be considered for the safety analysis.<br><br>For safety analysis the frequency tabulations of abnormal physical examination and abnormal clinical laboratory findings (≥5 UNL) will be presented for each treatment group. Summary statistics for clinical laboratory findings and vital signs will be presented. A list of concomitant medications taken during the study period will be summarised.<br><br>All adverse events seen during the study period will be listed.<br>All statistical analyses will be further described in the Statistical Analysis Plan.

ABBREVIATIONS

ADA	American Diabetes Association
AE	adverse event
ALP	alkaline phosphatase
ALT	alanine aminotransferase
Apo	Apoprotein
AST	aspartate aminotransferase
BMI	body mass index
BP	Blood pressure
BUN	blood urea nitrogen
CD4	Cluster of Differentiation 4
CHL	Cadila healthcare limited
CNS	central nervous system
CPK	creatinine phosphokinase
CRF	case report form
CVD	cardiovascular disease
CVS	cardiovascular system
DM	diabetes mellitus
ECG	Electrocardiogram
EEG	Electroencephalogram
FPG	fasting plasma glucose
GCP	Good Clinical Practice
GFR	glomerular filtration rate
GGT	Gamma glutamyl transpeptidase
GI	gastro-intestinal
HAART	highly active antiretroviral therapy
Hb	Haemoglobin
HbA1c	glycosylated haemoglobin
HCT	Hematocrit
HDL	high density lipoprotein
HIV	Human immunodeficiency virus
HOMA	Homeostasis model assessment
HOMA IR	Homeostasis model assessment for insulin resistance
hs-CRP	high sensitivity C-reactive protein
ICU	Intensive care unit
kcal	Kilocalorie
LDL	low density lipoprotein
MCH	mean corpuscular haemoglobin
MCHC	mean corpuscular haemoglobin concentration

MCV	mean corpuscular volume
MI	Myocardial infarction
OD	once daily
PI	Protease inhibitor
PPAR	peroxisome proliferator-activated receptors
RBC	red blood cell
RS	respiratory system
SAE	serious adverse event
TC	total cholesterol
TG	Triglyceride
TZD	Thiazolinedione
VLDL	very low density lipoprotein
WBC	white blood cell
ZRC	Zydus Research Centre

TABLE OF CONTENTS

CLINICAL TRIAL PROTOCOL SUMMARY 2
TABLE OF CONTENTS 12
1. INTRODUCTION 14
1.1 MEDICAL BACKGROUND 14
1.2 RATIONALE FOR CONDUCTING THE TRIAL 14
1.3 DRUG PROFILE 15
2. STUDY OBJECTIVES 16
2.1 GENERAL AIM / PRIMARY OBJECTIVE 16
2.2 PRIMARY ENDPOINT(S) 16
2.3 SECONDARY ENDPOINT(S) 16
3. STUDY POPULATION 17
3.1 NUMBER OF SUBJECTS PLANNED 17
3.2 CRITERIA(S) FOR INCLUSION AND EXCLUSION 17
3.4 WITHDRAWAL CRITERIA 18
4. TREATMENTS 20
4.1 TREATMENTS TO BE COMPARED 20
4.1.1 Investigational product 20
4.1.2 Comparator drugs(s) or intervention(s) 20
4.1.3 Dosage and treatment schedule 20
4.1.4 Packaging, labelling and re-supply 20
4.1.5 Storage conditions 21
4.2 CONCOMITANT THERAPY 21
4.2.1 Rescue medication and additional treatment(s) 21
4.2.2 Restrictions 21
4.3 TREATMENT COMPLIANCE 21
5. OBSERVATIONS 22
5.1 EFFICACY 22
5.1.1 Primary endpoint(s) 22
5.1.2 Secondary endpoint(s) 22
5.2 SAFETY 22
6. INVESTIGATIONAL PLAN 24
6.1 STUDY DESIGN AND PLAN 24
6.2 STUDY PROCEDURES AT EACH VISIT 24

6.3 STUDY SCHEDULES 24
6.3 ADHERENCE TO PROTOCOL 27
7. STATISTICS 28
7.1 POPULATION 28
7.1.1 Efficacy population 28
7.1.2 Safety population: 28
7.2 STATISTICAL ANALYSIS 28
7.2.1 Baseline Characteristics 28
7.2.2 Efficacy analysis 29
7.2.3 Safety Analysis 29
8. ADMINISTRATIVE MATTERS 31
8.1 ETHICS 31
8.1.1 Institutional Review Board or Independent Ethics Committee 31
8.1.2 Informed Consent and Subject Information 31
8.2 RECORDS 32
8.2.1 Drug Accountability 32
8.2.2 Emergency code break 33
8.2.3 Case Report Forms 33
8.2.4 Source documents 33
8.2.5 Direct access to source data / documents 34
8.3 QUALITY ASSURANCE AUDIT 35
8.4 PROCEDURES 35
8.4.1 Adverse Events 35
8.4.2 Emergency procedures 36
8.4.3 Pregnancy 36
8.5 RULES FOR AMENDING PROTOCOL 36
8.6 DISCONTINUATION OF THE TRIAL BY THE SPONSOR 37
8.7 STATEMENT OF CONFIDENTIALITY 37
8.8 PUBLICATION POLICY 37
9. REFERENCES 38

1. INTRODUCTION

1.1 MEDICAL BACKGROUND

The prognosis of HIV infection has drastically changed after the introduction of combination antiretroviral therapy often referred to as highly active antiretroviral therapy (HAART). But, lifelong exposure to HAART puts patients at a significant risk for long term metabolic adverse effects including lipodystrophy, insulin resistance, hyperlipidemia and increased cardiovascular morbidity.[1]

HIV lipodystrophy affects up to 50%-80% of patients receiving anti retroviral therapy. Patients with this condition develop a pattern of redistribution in body fat characterised by peripheral fat loss (facial and limb lipoatrophy) and central fat accumulation (buffalo hump, increased breast size and abdominal girth). Although linked to antiretroviral therapy, the exact etiology of HIV lipodystrophy remains unclear. The protease inhibitor (PI) class of anti-retrovirals, used in combination regimens for the treatment of HIV, has been associated with detrimental lipid changes. Recognised metabolic disturbances include abnormalities in both Triglyceride and cholesterol levels in the blood. Resultant cholesterol abnormalities tend to include elevation of LDL and VLDL concentrations.[2]

1.2 RATIONALE FOR CONDUCTING THE TRIAL

A variety of drugs have been studied to determine their influence on lipid profiles in patients suffering from HIV associated lipodystrophy due to HAART. Although hypolipidemic diets and physical exercise have also been found to have a role to play in the management of dyslipidemic HIV-infected patients, and are likely to be effective in this group, no randomised studies using these as monotherapy have been performed [2].

Recent research publications have shown the use of two lipid-lowering class of drugs, statins and fibrates, antiretroviral switching strategies and use of insulin-sensitising drugs. However, no single therapy is able to reach desirable clinical end point for HIV associated lipodystrophy. In order for firm management recommendations to be made in this setting, randomised trials are necessary [2].

ZYH1 is a novel predominately PPAR α agonist. It is expected to favorably modulate lipid and glucose profile and ameliorate insulin resistance as demonstrated in phase II studies with ZYH1. Thus, based on the above hypotheses and the results of phase II study, we now propose to undertake a prospective, multi-centric, open-label, single arm study to evaluate the safety and efficacy of 4mg of ZYH1 in hypertriglyceridemia in HIV associated lipodystrophy.

1.3 DRUG PROFILE

ZYH1, a dual PPAR agonist with predominantly PPARα and a moderate PPAR γ agonist activity, is (*S*)-α-Ethoxy-4-{2-[2-methyl-5-(4-methylthio)phenyl)]-1H-pyrrol-1-yl]-ethoxy})-benzenepropanoic acid magnesium salt.

For this study, safety and efficacy of 4mg of ZYH1 developed by Cadila Healthcare Ltd will be evaluated in hypertriglyceridemia in HIV associated lipodystrophy.

2. STUDY OBJECTIVES

2.1 GENERAL AIM / PRIMARY OBJECTIVE

To evaluate the safety and efficacy of 4mg of ZYH1 in hypertriglyceridemia in HIV associated lipodystrophy

2.2 PRIMARY ENDPOINT(S)

Percent change in TG levels from baseline to Week 6 and Week12.

2.3 SECONDARY ENDPOINT(S)

Percent change in the following parameters from baseline to Week 6 and Week 12.

- LDL
- VLDL
- HDL
- Total cholesterol
- Non HDL Cholesterol (Measured value)
- Apo (a)
- Apo B
- Fasting insulin and C-peptide for HOMA beta & IR

3. STUDY POPULATION

Subjects should be clinically diagnosed as HIV Lipodystrophy with confirmed diagnosis of HIV 1 and on highly active antiretroviral therapy (HAART) for at last 18 months with Triglyceride level ranging between 200 to 500 mg% .before getting enrolled in the trial.

3.1 NUMBER OF SUBJECTS PLANNED

The required sample size for this study to achieve 80% power at the 5% level of significance is a total of 50 subjects.

3.2 CRITERIA(S) FOR INCLUSION AND EXCLUSION

Inclusion Criteria (s):

•Age 18- 65 years

•Subjects should have confirmed diagnosis of HIV 1 and on highly active antiretroviral therapy (HAART) for at last 18 months.

•On stable ART regimen for at least 8 weeks prior to inclusion in the study and ART regimen not expected to change in next 3 months.

•Subjects clinically diagnosed as HIV Lipodystrophy (at least 1 moderate or severe Lipodystrophy feature identified by doctor and patient, except isolated abdominal obesity)

•Triglycerides between 200 – 500 mg%

•Subject has given informed consent for participation in this trial

• Subjects whose CD4 count is > $50/mm^3$

Exclusion Criteria(s):

- Subjects on insulin and/or glitazone / glitazar therapy
- Pregnancy and lactation
- Subjects with history of gall stone.

- Subjects with history of Cardiac failure.
- Subject with history of alcohol and/or drug abuse
- History of allergy, sensitivity or intolerance to the study drugs and their formulation ingredients.
- Active Opportunistic infection in last three months.
- History of malignancy or active neoplasm.
- Any active hormonal disease and/or hormonal treatment that may affect the outcomes of interest such as clinically overt hypo/hyperthyroidism, hypogonadism, hypercortisolism, or treatment with steroids or growth hormone.
- Hemoglobin below 9 g/dl or Total leucocyte count below 1000/mm^3 or Platelet count below 50,000/mm^3
- Subjects with history of myopathies or evidence of active muscle diseases or CPK ≥10 times UNL
- Subjects with history of active liver disease or hepatic dysfunction demonstrated by aspartate aminotransferase (AST) and alanine aminotransferase (ALT) ≥2.5 times of upper limits of normal or Bilirubin more than 2 times UNL
- Renal dysfunction (serum creatinine > 2 mg%)
- Participation in any other clinical trial in past 3 months

3.4 WITHDRAWAL CRITERIA

The investigator may withdraw a subject from the study for any of the following:

- Subject who reports serious AEs (SAE)
- Subject who develops any serious/severe diseases which requires admission in intensive care unit (ICU) or interventional surgery, even if it is not related with investigational drugs
- Any subject found to have entered the study in violation of this protocol or if the subject is uncooperative during the study.
- Any subject who interrupts the study medication for longer than 4 consecutive days.

- If it is felt in Investigator's / Medical Expert's opinion that it is not in the subject's best interest to continue.
- In case a subject becomes pregnant, then she will be withdrawn from the trial.
- Subject who do not report to the clinic on +/- 4 days as per the visit schedule.
- Subject's lack of compliance to the study protocol
- Any subject who requires the use of an unacceptable concomitant medication.
- Any subject who wishes to withdraw his / her consent for participation in the study.

Subjects who, after consenting to the study, decide not to take part after administration of the first dose of study medication will retain their number. The next subject enrolled will be given the next number.

The date the subject is withdrawn from the study and the reason thereof will be documented in the CRF.

4. TREATMENTS

ZYH1 4mg will be administered in hypertriglyceridemic patients with HIV associated lipodystrophy, once daily in the morning before breakfast, for a period of 12 weeks.

4.1 TREATMENTS TO BE COMPARED

4.1.1 Investigational product

ZYH1 4mg

4.1.2 Comparator drugs(s) or intervention(s)

NA

4.1.3 Dosage and treatment schedule

ZYH1 4mg will be given once orally in the morning before breakfast, for 12 weeks.

4.1.4 Packaging, labelling and re-supply

The Investigational drug will be packed, labelled and supplied by CHL. The investigator will confirm the receipt of investigational drug in writing.

All drug supplies must be stored in accordance with the manufacturer's instructions separately from normal hospital / practice stocks. Drug supplies, which will not be used, are to be returned to CHL after completion of the study. All supplies must be accounted at the end of the study period.

All the centers or investigators would be provided enough number of units of the investigational drug for the conduct of the entire study. An inventory management form should be filled up for the same at the investigator's site. Investigational drug would then be dispensed to each subject in such a way that the subject can take the dosages of the investigational drug in accordance with the protocol.

4.1.5 Storage conditions

4mg ZYH1 will be strored at room temperature.

4.2 CONCOMITANT THERAPY

1. Treatment of common illness will be allowed which shall be documented in the case report form (CRF).
2. Treatment of opportunistic infections will be allowed during study period. Same will be recorded in the CRF.

4.2.1 Rescue medication and additional treatment(s)

The investigator at his/her discretion will be allowed to offer a rescue medication for hypertriglyceridemia if required, however, such interventions shall be recorded in the CRF with description of type of intervention, dose and duration. If medication given is in violation to the protocol, then such subjects will be withdrawn from the trial.

4.2.2 Restrictions

1. Other lipid lowering drugs will not be allowed during the study.
2. Drugs affecting insulin resistance will not be allowed during the study.
3. Other drugs claimed for treatment of lipodystrophy will not be allowed during the study.
4. Change in antiteroviral treatment is not allowed during the study. If changes are necessary such patient will be withdrawn from study.

4.3 TREATMENT COMPLIANCE

The subjects will be asked to bring the container of the study medication on the next follow-up visit and compliance for dosing will be assessed by examination of the container and tablet count by study personnel out of the sight of the subject.

5. OBSERVATIONS

5.1 EFFICACY

5.1.1 Primary endpoint(s)

Percent change in TG levels from baseline to Week 6 and Week 12

5.1.2 Secondary endpoint(s)

Percent change in following parameters from baseline to Week 6 and Week 12.

- LDL
- VLDL
- HDL
- Total cholesterol
- Non HDL Cholesterol (Measured value)
- Apo (a)
- Apo B
- Fasting insulin and C-peptide for HOMA beta & IR

5.2 SAFETY

Following parameters will be assessed

- General and Systemic Clinical Examination: cardiovascular system (CVS), respiratory system (RS), gastrointestinal system (GI), central nervous system (CNS) etc.

- Laboratory Investigations: Complete blood count (CBC), aspartate transaminase (AST), alanine transaminase (ALT), alkaline phosphatase (ALP), total bilirubin, γ- glutamyl transpeptidase (GGT), Serum proteins, Blood urea nitrogen (BUN), Serum creatinine and Creatinine phosphokinase (CPK)

- Frequency and severity of adverse events (AEs) for all subjects enrolled will be recorded.

- All AEs, will be classified using
    - causality
    - severity
    - seriousness

6. INVESTIGATIONAL PLAN

6.1 STUDY DESIGN AND PLAN

A prospective, multi-centric, open-label, single arm study to evaluate the safety and efficacy of 4mg of ZYH1 in hypertriglyceridemia in HIV associated lipodystrophy.

6.2 STUDY PROCEDURES AT EACH VISIT

All the subjects will be evaluated as per the Inclusion & Exclusion Criteria and will undergo a thorough general and systemic examination and appropriate laboratory investigations to rule out other concomitant diseases prior to enrolment into the study and initiation of therapy.

6.3 STUDY SCHEDULES

Study schedule for the trial is given in Table 1.

Details of visit schedule:

Informed consent will be obtained before any trial related activity.

1) Visit 1, Screening/Enrolment Visit [Week ≥ -1 to 0]

- Subjects will be screened for the inclusion and exclusion criteria and those qualifying will be invited to participate in the study.
- Clinical evaluation will be done for baseline characteristics and anthropometry.
- After Clinical evaluations all baseline safety and efficacy parameters will be recorded as per Table 1.
- All laboratory investigations will be carried out after an overnight fasting.
- Enrolled Subjects will receive a study medication for next two weeks

2) Visit 2, [Week 2]

- Subjects will be clinically examined and given the study medications for next four weeks and also safety parameters will be assessed as per Table 1.
- Empty strips will be collected back by the investigators for compliance check.

3) Visit 3 [Week 6]

- Subjects will be clinically examined and given the study medications for next 6 weeks and also safety and efficacy parameters will be assessed as per Table 1.
- Empty strips will be collected back by the investigators for compliance check.

4) Visit 4 [Week 12]

- Subjects will be clinically examined and safety and efficacy parameters will be assessed as per Table 1.
- Empty strips will be collected back by the investigators for compliance check.

Unscheduled visits are allowed any time during the study for any untoward effect. Such visits will be recorded in the case record forms (CRFs).

If further investigations are required in case of any AE, investigator will assess the AE and take necessary action, if required. Subjects will be advised to contact the investigator for any complaints within the next two weeks.

During the above period, if any subject misses the drug administration up to 3 days, it will not be considered drop-out or protocol deviation.

During the 12 week program, a designated person from the centre will preferably interview the subject for his/her general health, telephonically.

Table 1: Visit and Investigation Schedule

Activity	Screening / Enrolment Visit 1 (Wk ≥-1 to 0)	Visit 2 (Week 2)	Visit 3 (Week 6)	Visit 4 (Week 12)
Demographics	✓			
Inclusion / Exclusion criteria	✓			
Informed Consent	✓			
Medical History	✓	✓	✓	✓
Vital Signs & Physical Examination	✓	✓	✓	✓
Efficacy studies:<br>Laboratory:<br>1. Triglyceride<br>2. LDL<br>3. VLDL<br>4. HDL<br>5. Total cholesterol<br>6. Non HDL Cholesterol<br>7. Apo(a)<br>8. Apo B<br>**9.** Fasting insulin and C-peptide for HOMA beta & IR	✓		✓	✓
Laboratory studies(safety)<br>1. CBC<br>2. LFT<br>3. RFT<br>4. CPK	✓	✓	✓	✓
ECG	✓			✓
Pregnancy test for female subjects (advise for contraception)	✓			
Dispensing of Study Medication	✓	✓	✓	
Study Medication Tablet Count		✓	✓	✓
Recording of Adverse Events		✓	✓	✓
Global Tolerability Assessments				✓
Study Completion				✓

6.3 ADHERENCE TO PROTOCOL

Investigator shall strictly adhere to the protocol and GCP guidelines.

All subjects will be strictly required to follow the instructions given to them as per this protocol. For any deviation or violation from protocol, considered serious by investigator, the subject may be withdrawn from the trial. [Section 3.4].

7. STATISTICS

7.1 POPULATION

7.1.1 Efficacy population

7.1.1.1 Intention-to-treat population (ITT):

The intention-to-treat (ITT) population will be the set of all subjects who receive study medication and have at least one post baseline efficacy data available.

7.1.1.2 Per-protocol population (PP):

The per-protocol population (PP) will be the set of all subjects who receive study medication and complete Week 12 of the study in compliance to the protocol.

The PP analysis will be considered as definitive, while the ITT analysis will be considered as supportive during the trial analysis.

7.1.2 Safety population:

The safety population will be the set of all subjects who receive at least 1 dose of either study medication.

7.2 STATISTICAL ANALYSIS

7.2.1 Baseline Characteristics

The demographic and baseline characteristics will be summarized. For continuous measurements such as age, the mean, median, standard deviation and range will be tabulated. For categorical measurements such as gender, the frequencies will be computed.

7.2.2 Efficacy analysis

The primary efficacy variable is the reduction in Triglyceride (TG) at Week 6 and 12 of the treatment period compared with baseline. The change from baseline will be determined as the difference between the means for the treatment period (Weeks 6 and 12) and the baseline.

For the efficacy endpoints, treatment effect will be evaluated using an analysis of variance (ANOVA) model with factors for baseline and treatment. Treatment effects will be estimated using the least-square means and 95% confidence intervals (CIs) from the ANOVA model. Statistical significance will be defined as a two-sided p-value <0.05.

All other secondary endpoints would be analysed using appropriate statistical methods.

Intent-to-treat (ITT) and/or Per Protocol (PP) analysis will be carried out for the study trial. The PP analysis will be considered definitive while the ITT analysis will be considered supportive during the trial analysis. All the subjects enrolled in the study will be considered for the safety analysis.

7.2.3 Safety Analysis

For safety analysis the frequency tabulations of abnormal physical examination and abnormal clinical laboratory findings (≥5 UNL) will be presented for each treatment group. Summary statistics for clinical laboratory findings and vital signs will be presented. A list of concomitant medications taken during the study period will be summarised.

All adverse events seen during the study period will be listed.

All statistical analyses will be further described in the Statistical Analysis Plan.

8. ADMINISTRATIVE MATTERS

The trial will be carried out in compliance with the protocol, the principles laid down in the Declaration of Helsinki, in accordance with the ICH Harmonised Tripartite Guideline for Good Clinical Practice (GCP) and in accordance with applicable regulatory requirements.

8.1 ETHICS

8.1.1 Institutional Review Board or Independent Ethics Committee

The trial will not be initiated before the protocol and informed consent and subject information form have been reviewed and received approval / favourable opinion from the local Institutional Review Board (IRB) or an Independent Ethics Committee (IEC). Should a protocol amendment be made that needs IRB / IEC approval, the changes in the protocol will not be instituted until the amendment and revised informed consent (if appropriate) has been reviewed and received approval / favourable opinion from the local IRB or IEC. A protocol amendment intended to eliminate an apparent immediate hazard to subjects may be implemented immediately providing that the appropriate regulatory authorities and IRB / EC are notified as soon as possible and an approval is requested. Protocol amendments only for logistical or administrative changes may be implemented immediately; the IRB /IEC need to be informed only.

The constitution of the IRB or IEC must meet the requirements of the regulatory agencies. A list of the IRB / IEC members, with names and qualifications, will be requested. If such a list is unavailable, the investigator must provide the name and address of the IRB/IEC along with a statement from the IRB/IEC that it is organised according to GCP and the applicable laws and regulations. The IRB or IEC must also perform all duties outlined by the requirements of the regulatory agencies.

8.1.2 Informed Consent and Subject Information

Prior to subject participation in the trial, written informed consent will be obtained from each subject according to the regulatory and legal requirements of the participating country. Each signature must be dated by each signatory and the

informed consent and any additional subject information form retained by the investigator as part of the study records. A signed copy of the informed consent and any additional subject information must be given to each subject or the subject's legally authorised representative.

The subject must be informed that his / her medical records may be examined by authorised monitors or Clinical Quality Assurance auditors appointed by Cadila Healthcare Limited, by appropriate IEC / IRB members and by inspectors from regulatory authorities.

Should a protocol amendment be made, the subject consent form and subject information form may need to be revised to reflect the changes to the protocol. It is the responsibility of the investigator to ensure that an amended consent form is reviewed and received approval / favourable opinion from the IRB or IEC, and that it is signed by all subjects subsequently entered in the trial and those currently in the trial, if affected by the amendment.

8.2 RECORDS

8.2.1 Drug Accountability

Drug supplies, which will be provided by the sponsor, must be kept in a secure, limited access storage area under the storage conditions defined by the sponsor. If necessary, a temperature log must be maintained to make certain that the drug supplies are stored at the correct temperature.

The investigator and/or pharmacist must maintain records of the product's delivery to the trial site, the inventory at the site, the use by each subject, and the return to the sponsor or alternative disposition of unused product(s). These records will include dates, quantities, batch/serial numbers, expiration dates (if applicable), and the unique code numbers assigned to the investigational product(s) and trial subjects. Investigators will maintain records that document adequately that the subjects were provided the doses specified by the protocol and reconcile all investigational product(s) received from the sponsor. At the time of return to the sponsor, the investigator must verify that all unused or partially used drug supplies have been returned by the clinical trial subject and that no remaining supplies are in the investigator's possession.

8.2.2 Emergency code break

This will not be applicable as the study is an open-label study.

8.2.3 Case Report Forms

Case report forms for individual subjects will be provided by the sponsor. A copy remains with the investigator as a permanent record, and the original will be returned to the sponsor.

Case report forms are used to record clinical trial data and are an integral part of the trial and subsequent reports. The case report forms, therefore, must be legible and complete. All forms must be filled in using a black ballpoint pen. Errors must be lined out but not obliterated and the correction inserted, initialled and dated.

The investigator must sign a declaration ensuring accuracy of data recorded in the case report forms.

Case reports forms must be kept current to reflect subject status at each phase during the course of trial. Subjects are not to be identified on the case report form by name. Appropriate coded identification (e.g. Subject Number) and subject initials must be used. The investigator must make a separate confidential record of these details (subject identification code list) to permit identification of all subjects enrolled in a clinical trial in case follow-up is required.

Relevant medical history prior to enrolment will be documented at the baseline visit. Thereafter during the trial narrative statements relative to the subject's progress during the trial will be maintained. See also 8.2.4.

The investigator will be responsible for retaining all records pertaining to the trial as specified in the agreement.

8.2.4 Source documents

Source documents provide evidence for the existence of the subject and substantiate the integrity of the data collected. Source documents are filed at the investigator's site. Data reported on the Case Report Forms that are derived from source

documents must be consistent with the source documents or the discrepancies must be explained.

The investigator may need to request previous medical records or transfer records, depending on the trial; also current medical records – not just shadow charts – must be available.

The following data to be reported on the CRF should be included and derived from the source documents:

- Subject identification (initials, gender, data of birth/age)
- Subject participation in the trial (substance, trial number, subject number, date informed consent given)
- Dates of subject's visits
- Medical history
- Medication history
- Adverse events (AE onset and end)
- Serious adverse events (SAE onset and end)
- Originals or copies of laboratory results
- Originals or copies of X-rays and Ultrasound findings, ECG results, EEG results, neurological findings, endoscopy findings and other results based on hard copies of medical machinery, etc., if applicable.
- Conclusion of subject's participation in the trial.

8.2.5 Direct access to source data / documents

The investigator / institution will permit trial related monitoring, audits, IRB / IEC review and regulatory inspection, providing direct access to all related source data / documents. Case report forms and all source documents, including progress notes and copies of laboratory and medical test results must be available at all times for

review by the sponsor's clinical trial monitor and inspection by health authorities (e.g., FDA, DCGI, etc.). The CRA/on-site monitor may review all case report forms, and written informed consents. The accuracy of the data will be verified by reviewing the documents described in Section 8.2.4.

All the ECG's taken at the centre will be photocopied and provided to the sponsor.

8.3 QUALITY ASSURANCE AUDIT

A quality assurance audit of this trial may be conducted by the sponsor or sponsor's designees. The quality assurance auditor will have access to all medical records, the investigator's trial related files and correspondence, and the informed consent documentation that is relevant to this clinical trial.

8.4 PROCEDURES

8.4.1 Adverse Events

All adverse events occurring during the course of the clinical trial (i.e., from signing the informed consent onwards) will be collected, documented and reported to the sponsor by the investigator according to the specific definitions and instructions detailed in the "Adverse Event Reporting" section of the Investigator Site Master File.

An adverse event is defined as any untoward medical occurrence in a clinical investigation subject administered a pharmaceutical product and which does not necessarily have to have a causal relationship with this treatment.

A serious adverse event is defined as any adverse event which results in death, is immediately life-threatening, results in persistent or significant disability / incapacity, requires or prolongs subject hospitalisation, is a congenital anomaly / birth defect, is to be deemed serious for any other reason representing a significant hazard, which is comparable to the aforementioned criteria.

All adverse events, serious and non-serious, will be fully documented on the appropriate case report form(s). For each adverse event, the investigator will provide the onset, duration, intensity, treatment required, outcome and action taken with the investigational product. The investigator will determine the relationship of the

investigational product to all adverse events as defined in the “Adverse Event Reporting” Section of the Investigator Site Master File.

The investigator has the responsibility to report adverse events occurring in a period after a subject completes the trial, as defined and described in the “Adverse Event Reporting” section of the Investigator Site Master File.

8.4.2 Emergency procedures

Any serious or significant adverse event, whether or not considered related to the investigational product, and whether or not the investigational product has been administered, must be reported immediately by telephone / fax to the sponsor. Details regarding this reporting procedure are provided in the Investigator Site Master File.

Following all such telephone / fax reports the Clinical Monitor must provide a written report of the adverse event and any sequel to the Drug Safety Officer of the sponsor according to the appropriate SOP. These narratives, which confirm the information collected by telephone and may give additional information not available at the time of the initial report.

8.4.3 Pregnancy

At screening every female subject will be investigated for urine pregnancy test. Women should not become pregnant during the trial and for at least 1 month after the end of the trial period. Adequate contraceptive measures should be taken to prevent pregnancy. Even when contraceptive methods are used, there is a small risk that pregnancy might occur. In case a subject becomes pregnant, then she will be withdrawn from the trial.

8.5 RULES FOR AMENDING PROTOCOL

All amendments must be documented, dated and signed by all signatories (or their successors) of the original protocol.

8.6 DISCONTINUATION OF THE TRIAL BY THE SPONSOR

Cadila Healthcare Limited reserves the right to discontinue this trial at any time for failure to meet expected enrolment goals, for safety or any other administrative reasons. The investigator will be reimbursed for reasonable expenses incurred if it is necessary to terminate the trial as per the agreement.

8.7 STATEMENT OF CONFIDENTIALITY

Individual subject medical information obtained as a result of this study is considered confidential and disclosure to third parties is prohibited with the exceptions noted below. Subject confidentiality will be further ensured by utilising subject identification code numbers to correspond to treatment data in the computer files.

Such medical information may be given to the subject’s personal physician or to other appropriate medical personnel responsible for the subject’s welfare.

Data generated as a result of this trial are to be available for inspection on request by the participating physicians, the sponsor’s representatives, by the IRB or IEC and the regulatory health authorities.

8.8 PUBLICATION POLICY

Cadila Healthcare Limited is as much as possible dedicated to support process of free exchange of relevant scientific information. Any publication of the result of this trial must be consistent with the Cadila Healthcare Limited publication policy. The rights of the investigator and of the sponsor with regard to publication of the results of this trial are described in the investigator agreement.

9. REFERENCES

1. Jussi Sutinen. The effects of Thiazolidinediones on Metabolic Complications and Lipodystrophy in HIV-Infected patients, *PPAR Research,* Volume 2009, Article ID 373524.

2. C.McGoldrick; C.L.S.Leen. The management of dyslipidemias in Antiretroviral-treated HIV Infection: A Systematic Review; HIV Med.2007; 8 (6); 325-334.
