# Supplementary material for: A Prospective, Multicentre, Open-Label Single-Arm Exploratory Study to Evaluate Efficacy and Safety of Saroglitazar on Hypertriglyceridemia in HIV Associated Lipodystrophy
Source: PLoS One. 2016 Jan 20;11(1):e0146222. doi: 10.1371/journal.pone.0146222 (PMC4720399; doi:10.1371/journal.pone.0146222)
Supplement: S2 File — (PDF) [file pone.0146222.s002.pdf]

(Government of Maharashtra)

Institutional Ethics Committee

Department of Pharmacology,
Grant Medical College & Sir J.J. Group of Hospitals,
Byculla, MUMBAI 400 008
Ph: +91-22-2373 5555 Extn. 2322

No.IEC/Pharm/648 /2010, Dt.03/08/2010.

To,
Dr.Alaka K. Deshpande,
Principal Investigator,
Professor of Medicine,
Grant Medical College &
Sir J.J.Group of Hospitals,
Mumbai.

Dear Dr.

The Institutional Ethics Committee of Grant Medical College and Sir J.J.Group of Hospital, Mumbai, reviewed and discussed your application of Research Project titled "A prospective, multi-centric, open label, single arm study to evaluate the safety and efficacy of 4mg of ZYH1 in hypertriglyceridemia in HIV associated lipodystrophy".

The documents submitted by you vide your letter No.AKD/Res.Proj/133/10, dated 3rd August, 2010 were reviewed by the following Institutional Ethics Committee members by circulation :-

Members Present	Qualification	Affiliation/Designation within EC
1) Dr.Danial Joseph	M.D. Pharmacology	Professor of Pharmacology, Mahatma Gandhi Medical College, Navi Mumbai. **Chairman, IEC.**
2) Shri U.R.Patil	B.A., L.L.B.	Hon'ble Retd., Judge, **Member, IEC.**
3) Dr.D.A.Palande	M.Ch. (Neurosurgery)	Professor of Neurosurgery, Grant Medical College, Mumbai. **Member, IEC.**

4) Dr.A.D.Rathod	M.D. Paediatric<br>D.M. Cardiology.	Professor & HOD Paediatric,<br>Grant Medical College,<br>Mumbai.<br>**Member, IEC.**
5) Dr.M.B.Tayade	M.S. Gen.Surgery	Professor & HOD Surgery,<br>Grant Medical College and<br>Sir J.J.Group of Hospitals,<br>Mumbai.<br>**Member, IEC.**
6) Dr.P.B.Sawant	M.D. (P.S.M.)	Associate Professor of P.S.M.,<br>Grant Medical College,<br>Mumbai.<br>**Member, IEC.**
7) Mrs.F.M.Merchant	B.Com.	Social Worker,<br>**Member, IEC.**
8) Shri Eknath Thakur	S.S.C.	Lay Person.<br>**Member, IEC.**
9) Dr.S.B.Patel,	M.D.Pharmacology.<br>D.C.H. Paedicatics.	Professor & Head<br>Department of Pharmacology,<br>Grant Medical College,<br>Mumbai.<br>**Member Secretary, IEC.**

We approve the trial to be conducted in its presented form.

The Institutional Ethics Committee expects to be informed about the progress of the study, any SAE occurring in the course of the study, any changes in the protocol. Patient should not be put to any financial hardship and subject information/informed consent to be taken and ask to provide a copy of the final report.

The Institutional Ethics Committee functions based on GCP and ICMR Guidelines.

Yours Sincerely,

(Dr.S.B.Patel),
Member Secretary
Institutional Ethics Committee &
Prof. and Head Department of Pharmacology,
Grant Medical College, Mumbai.
