# Supplementary material for: A Prospective, Multicentre, Open-Label Single-Arm Exploratory Study to Evaluate Efficacy and Safety of Saroglitazar on Hypertriglyceridemia in HIV Associated Lipodystrophy
Source: PLoS One. 2016 Jan 20;11(1):e0146222. doi: 10.1371/journal.pone.0146222 (PMC4720399; doi:10.1371/journal.pone.0146222)
Supplement: S3 File — (PDF) [file pone.0146222.s003.pdf]

Independent Ethics Committee-Aditya

ACEAS™ Clinical Research

001, Aradhya Apartments, Behind Hero-Honda Show Room, Under New Shreyas Fly Over, Ambawadi, Ahmedabad 380 015. India.

Telephones : +91 79 26460930, Mobile : +91 93280 18777 / 9327924927, Email : aceas@rediffmail.com • www.aceas.co.in

Dr. Harsh Toshniwal
IDTM Clinic, 402
Gala Business Centre
Near Rajhans Society
Saint Xavior's Commerce College Road
Off C.G. Road, AHMEDABAD 380 006

25-June-2010

Dear Dr. Toshniwal,

Subject: Review and approval of clinical trial Protocol No. ZYH1.09.003.01.PROT titled "A prospective, multi-centric, open-label, single arm study to evaluate the safety and efficacy of 4 mg of ZYH1 in hypertriglyceridemia in HIV associated lipodystrophy"

This has reference to your request for review and approval of your participation in the above multi-centric clinical study, being sponsored and organized by Cadila Healthcare Limited, Ahmedabad.

Independent Ethics Committee-Aditya (IEC-Aditya) has reviewed the protocol and other required documents for the above clinical study in its meeting held on Friday, 25-June-2010, in which the following members participated:

1	Dr. A.K. Batham
2	Mrs. Sanchita Mitra
3	Dr. Jayesh V. Trivedi
4	Mr. Rajendra D. Rajput

5	Dr. Jagdish P. Joshipura
6	Dr. S.D. Biswas
7	Dr. V. M. Khandhar

Following documents, as per the regulatory requirements, have been reviewed:

Documents	Version/No.	Date of document
Investigator's Brochure	Version 03	10-November-2008
Protocol No. ZYH1.09.003.01.PROT	Version 1.0	09-March-2009
Amendment Sheet, Protocol No. ZYH1.09.003.01.PROT	-	02-November-2009
Case Report Form	Version 1.0	09-March-2009
Informed Consent Document including Informed Consent Form ZYH1.09.003.01.PROT in English	Version 1.0	09-March-2009
Permission of Drugs Controller General (India)	F.No.12-05/05-DC	16-June-2010
Your undertaking to conduct the study	-	19-May-2009
Your CV	-	19-May-2009
Clinical Trial Insurance For the period-20/03/2009 to 19/03/2010	141500/48/2009/3914	17-March-2009

INDEPENDENT ETHICS COMMITTEE-ADITYA

We understand, the sponsor has communicated your name, as one of the investigators for this multi-centric clinical study, to the Office of the Drugs Controller General (India), New Delhi.

Copy of the votes of the members of the IEC-Aditya who participated in the meeting are attached herewith for confirmation and record. Based on the favorable opinion given by all the 7 out of 7 members who participated in the meeting, Independent Ethics Committee – Aditya is pleased to grant approval to the conduct of the reviewed clinical study by you at, Dr. Harsh Toshniwal, IDTM Clinic, 402, Gala Business Centre, Near Rajhans Society, Saint Xavior's Commerce College Road, Off C.G. Road, AHMEDABAD 380 006, subject to the following conditions:

i. the clinical study will be conducted in full compliance to the regulatory directives indicated in the letter from the Office of the Drugs Controller General (India), New Delhi,
ii. the IEC-Aditya shall do a continuing review of the progress of the trial for which you shall provide periodic clinical updates on the progress of the study,
iii. the IEC-Aditya shall provide, according to the applicable regulatory requirements, expedited review and approval/favorable opinion on minor change(s) in the trial during the course of its conduct, if required,
iv. that no deviations from, or changes of, the protocol would be initiated without prior written IEC-Aditya approval/favorable opinion of an appropriate amendment, except when necessary to eliminate immediate hazards to the subjects or when the change(s) involves only logistical or administrative aspects of the trial (e.g., change of monitor(s), telephone number(s))
v. that the investigator would promptly report to the IEC-Aditya : (a) deviations from, or changes in, the protocol to eliminate immediate hazards to the trial subjects, (b) changes increasing the risk to subjects and/or affecting significantly the conduct of the trial, (c) all adverse events including, serious and unexpected, and (d) new information that may adversely affect the safety of the subjects or the conduct of the trial, and
vi. that the IEC-Aditya would promptly notify in writing the investigator/institution concerning: (a) its trial-related decisions/opinions, (b) the reasons for its decisions/opinions, and (c) procedures for appeal on its decisions/opinions.

Best regards,

A. K. Batham, M.D., 25 Jun. 10
Chairman,

Independent Ethics Committee-Aditya
001 Aradhya Apartments,
Behind Hero-Honda Show Room,
Under New Shreyas Fly Over,
Ambawadi, Ahmedabad-380015. India

ndependent Ethics Committee-Aditya

.CEAS™ Clinical Research

001, Aradhya Apartments, Behind Hero-Honda Show Room, Under New Shreyas Fly Over, Ambawadi, Ahmedabad 380 015. India.
Telephones : +91 79 26460930, Mobile : +91 93280 18777 / 9327924927, Email : aceas@rediffmail.com • www.aceas.co.in

VOTING SHEET

Cadila Healthcare Limited, Ahmedabad

25-June-2010,Time: 07:00 pm

Project No.: ZYH1.09.003.01.PROT **Version No.:** 1.0, Dated 09-March-2009

Clinical Study: A prospective, multi-centric, open-label, single arm study to evaluate the safety and efficacy of 4 mg of ZYH1 in hypertriglyceridemia in HIV associated lipodystrophy

Clinical Investigator: Dr. Harsh Toshniwal, IDTM Clinic, 402, Gala Business Centre, Near Rajhans Society, Saint Xavior's Commerce College Road, Off C.G. Road, AHMEDABAD 380 006

No.	Member	Approvable	Non-approvable	Modifications required for approval
1	Dr. A.K. Batham	[illegible]	–	–
2	Dr. Avani F. Amin		–	–
3	Dr. Vinod M. Khandhar	Khandhar VM	–	–
4	Mrs. Sanchita Mitra	[illegible]	–	–
5	Dr. Swapna D.Biswas	[illegible]	–	–
6	Dr. Jagdish. P. Joshipura	[illegible]	-	.
7	Dr. Jayesh V. Trivedi	[illegible]	–	–
8	Mr. Rajendra D. Rajput	[illegible]	–	–

IEC-Aditya
